# A compact quad-port dielectric resonator MIMO antenna with intrinsic isolation for sub-6 GHz 5G network

**DOI:** 10.1038/s41598-026-47167-5

**Published:** 2026-05-11

**Authors:** Arpita Patel, Trushit Upadhyaya, Rajat Pandey, Upesh Patel, Yu-Jen Chi, Om Prakash Kumar, M. V. Swati

**Affiliations:** 1 Electronics & Communication Department, Chandubhai S. Patel Institute of Technology, Charotar University of Science & Technology, Changa, Gujarat India; 2 Electronics and Communication Department, Government Engineering College, Gandhinagar, Gujarat India; 3 Department of Electrical and Computer Engineering, Tamkang University, New Taipei City, Taiwan; 4 Manipal Institute of Technology, Manipal Academy of Higher Education, Manipal, 576104 India; 5 Department of Electronics and Communications Engineering, National Institute of Technology (NIT) Silchar, Silchar, 788010 Assam India

**Keywords:** Dielectric resonator antenna (DRA), Quad-port MIMO antenna, Sub-6 GHz 5G, Dual-band, Intrinsic isolation, SDG 9 (Industry, innovation and infrastructure), SDG 11 (Sustainable cities and communities), SDG 12 (Responsible consumption and production), Engineering, Physics

## Abstract

This paper presents the systematic design, fabrication, and experimental validation of a compact quad-port dielectric resonator antenna (DRA)–based MIMO system for sub-6 GHz 5G applications. An aperture-fed excitation combined with optimized spatial arrangement is employed to achieve high inter-port isolation intrinsically, without using additional parasitic decoupling networks or external isolation elements. Each radiating element supports dual-mode operation by exciting a fundamental linearly polarized TE_11_δ mode at 3.5 GHz and a higher-order TE_12_δ mode at 3.9 GHz, enabling dual-band performance. The proposed four-port MIMO antenna achieves inter-port isolation exceeding 20 dB at the operating frequencies of 3.5 GHz and 3.9 GHz within a compact footprint of 0.35λ₀ × 0.35λ₀. Experimental results demonstrate excellent diversity characteristics with ECC below 0.05, CCL of approximately 0.1 bps/Hz, peak gain up to 2.62 dBi, and radiation efficiency approaching 89%. The moderate gain level is well suited for compact user terminals, indoor access points, and small-cell 5G devices, where omnidirectional coverage and low correlation are prioritized over high directional gain. The close agreement between simulated and measured results confirms the robustness of the design, making it a promising candidate for compact sub-6 GHz 5G communication devices.

## Introduction

The demand for faster and more reliable wireless communication has never been greater. With the rapid growth of 5G networks, antennas must now support high data rates, robust connectivity, and wide-area coverage. Multiple-Input Multiple-Output (MIMO) has become indispensable in meeting these requirements as it uses spatial multiplexing and diversity to overcome multipath fading and improve channel capacity^1,2^. In this context, the proposed antenna is specifically intended for compact user equipment, indoor access points, and small-cell terminals, where moderate gain combined with high isolation and polarization diversity is prioritized over high directional gain. However, integrating several antennas into compact devices is far from straightforward. One of the central design challenges is achieving strong isolation between antenna ports without increasing the system’s size^3,4^. Insufficient isolation leads to correlation between channels and ultimately degrades the diversity performance that MIMO is intended to provide^5,6^.

A number of techniques have been explored to address this issue. Designers often introduce additional decoupling features such as defected ground structures (DGS)^9^, etched slots in the ground plane or radiators^8^, or even parasitic elements and neutralization lines^10^. While these solutions can work, they frequently come with trade-offs. They can complicate the geometry, limit impedance bandwidth, or make the antenna more sensitive to fabrication tolerances. More importantly, many reported designs struggle to deliver isolation levels higher than approximately 15–17 dB while also supporting desirable characteristics, such as dual-band behaviour and stable radiation performance^11,15^.

Several examples from the literature illustrate both the progress and the limitations of existing methods. For instance, a four-by-four hexagonal patch MIMO antenna achieves dual-band response through slot etching, with ground plane modification used to improve isolation^8^. A different design employs a rectangular dielectric resonator with a circular aperture and an S-shaped ground plane for Sub-6 GHz operation^9^. Similarly, an inverted-F antenna with a broad T-slot is proposed for WLAN/5G systems^10^, while other studies have looked at modified L-shaped^11^, arc-shaped^12^, dipole-based^13^, and uniplanar^14^ MIMO arrays. Additional work has focused on dual-port hexagonal ring-based radiators^15^. In parallel, the dielectric resonator antenna (DRA) community has reported composite rectangular^17^, ring-based^18,19^, and cylindrical^20^ MIMO designs aimed at WLAN, WiMAX, & Sub-6 GHz applications. More recently, studies have also demonstrated improved isolation in aperture-fed DR-MIMO antennas^21^, self-isolated compact MIMO structures^22^, and novel integration strategies for advanced RF systems^23,24^, further broadening the application scope of dielectric resonator-based designs. Collectively, these studies highlight the diversity of techniques available; yet, they also make clear that combining compactness, high efficiency, and strong isolation remains a challenging task.

Dielectric resonator antennas (DRAs) are particularly attractive for addressing these challenges due to their high radiation efficiency and flexible modal excitation capability. Unlike traditional patch antennas, which often suffer from reduced efficiency and bandwidth when fabricated on low-cost substrates, DRAs inherently offer higher radiation efficiency since they are free from conductor losses. They also provide wide impedance bandwidth and enable excitation of multiple resonant modes through different feeding techniques^17^. This modal flexibility can support pattern diversity and multimode operation, which are valuable characteristics for MIMO systems operating in dense scattering environments. Furthermore, the reduced surface-wave propagation in DRAs helps suppress mutual coupling in multi-port configurations, making them well suited for compact MIMO implementations. Despite several reported DRA-based MIMO studies^21–24^, achieving a balance between compact size, high isolation, and dual-band operation without employing additional decoupling structures remains a significant challenge. In addition, several studies have explored circularly polarized dielectric resonator antenna (CP-DRA) configurations for wireless communication and wideband applications^25,26^. Conventional aperture-fed DRA MIMO designs often experience increased mutual coupling when antenna elements are placed in close proximity, which typically necessitates additional decoupling techniques. In contrast, the proposed antenna addresses this limitation through controlled modal excitation and optimized spatial arrangement, thereby enabling intrinsic isolation without the use of extra decoupling elements. The design makes use of two distinct resonant modes within a single dielectric resonator: the fundamental TE₁₁δ mode at 3.5 GHz and a higher-order TE₁₂δ mode at 3.9 GHz. This dual-mode operation enables dual-band coverage suitable for sub-6 GHz 5G and WiMAX applications, while simultaneously improving port isolation through a combination of spatial and modal diversity. In this work, the term intrinsic isolation refers to isolation achieved without the use of separate parasitic or lumped decoupling networks, relying instead on antenna geometry, modal diversity, and optimized spatial arrangement.

Key outcomes of this study include a compact quad-port DRA-MIMO antenna operating simultaneously at 3.5 GHz (fundamental TE₁₁_δ_ mode) and 3.9 GHz (higher-order TE₁₂_δ_ mode), achieving isolation above 20 dB through modal and spatial optimization, along with excellent diversity performance (ECC < 0.05, radiation efficiency up to 89%, channel capacity loss 0.1 bps/Hz).

Unlike most reported Sub-6 GHz DRA-based MIMO systems that rely on defected ground structures, parasitic elements, or neutralization lines to enhance isolation, the proposed design achieves isolation exceeding 20 dB purely through controlled modal excitation and spatial configuration. Furthermore, while many existing DRA-MIMO designs operate in single-mode or single-band configurations, the present work intentionally excites both the fundamental TE₁₁_δ_ mode and a higher-order TE₁₂_δ_ mode within a compact quad-port arrangement. This controlled multi-mode operation enables dual-band functionality without increasing structural complexity. The combination of compact footprint (65 × 65 mm²), intrinsic isolation, low ECC (< 0.05), and minimal CCL (0.1 bps/Hz) distinguishes the proposed antenna from prior Sub-6 GHz DRA MIMO implementations.

The following sections are organized accordingly. Section  2 describes antenna modeling and configuration. Sections  3 and 4 present measured and simulated results, including a detailed discussion of MIMO diversity parameters. The fifth section highlights how the presented design measures up against state-of-the-art antenna solutions, and Sect.  6 provides the conclusion.

## Antenna design configuration

**Fig. 1:**
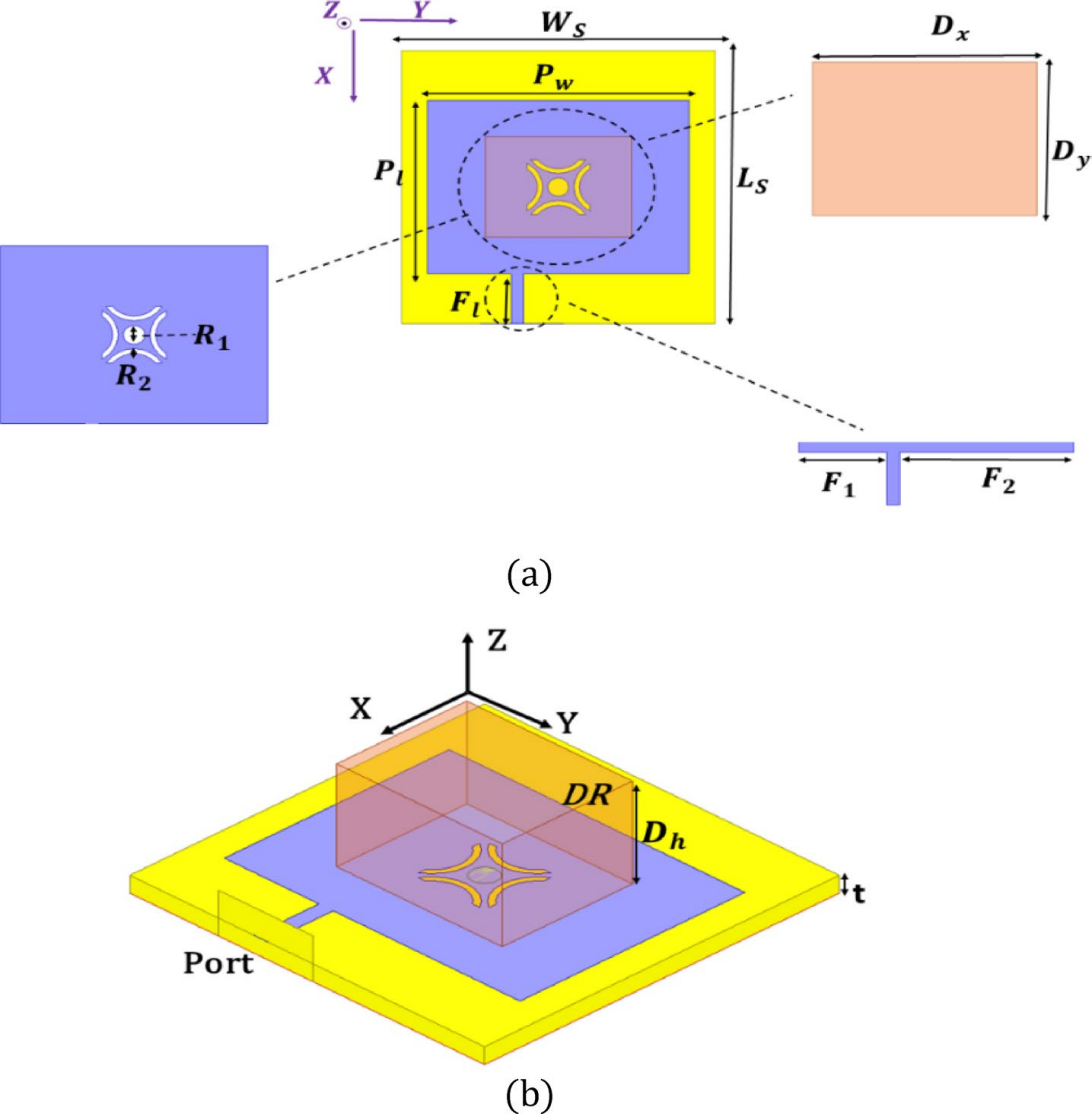
Designed DR antenna (**a**) Plan view (**b**) 3D view.

**Fig. 2:**
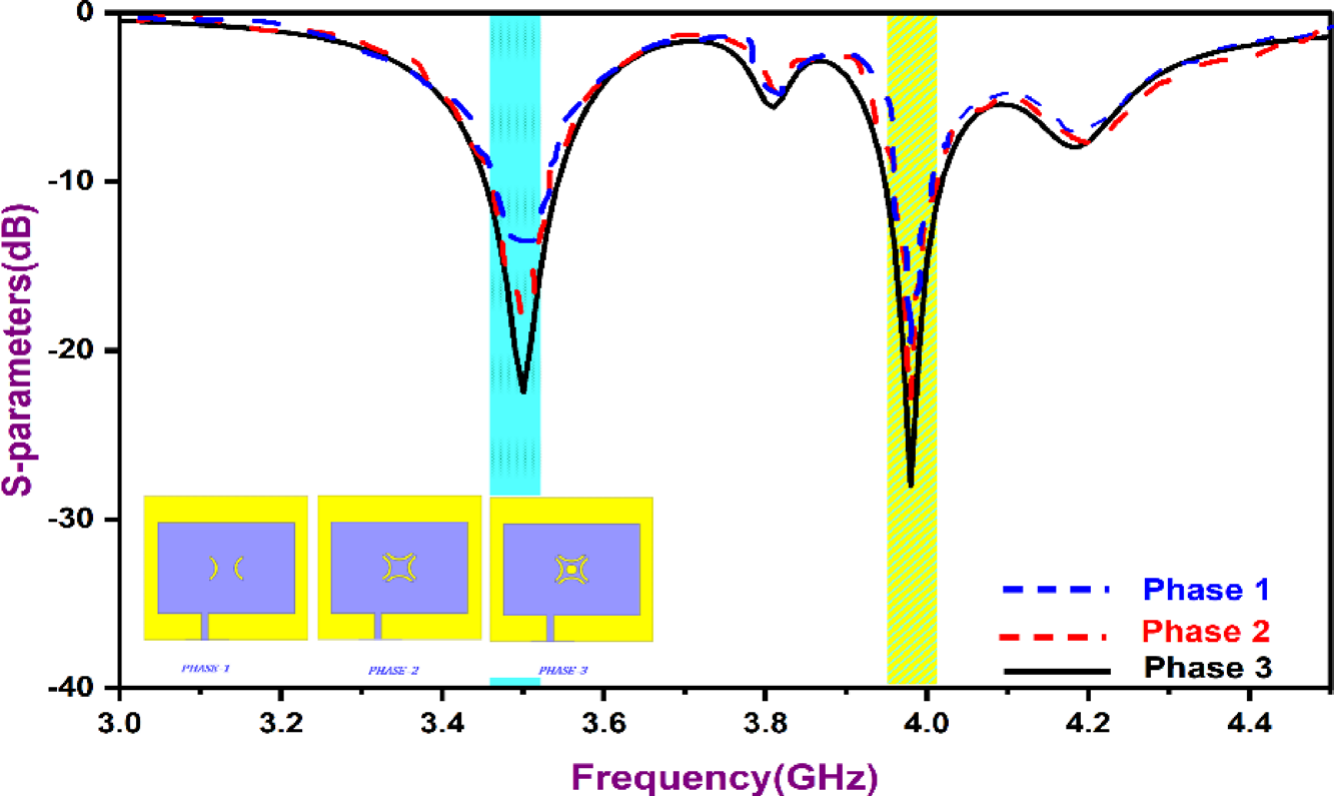
Optimization of aperture slots for feed coupling.

Figure 1 depicts the DR-antenna that has been designed. The proposed antenna employs a rectangular dielectric resonator (DR) positioned on a grounded substrate of dimensions 30 mm × 30 mm with a thickness of 1.56 mm. The dielectric resonator has physical dimensions D_x_ × D_y_ × D_h_ = 11 mm × 14 mm × 9 mm, as summarized in Table 2. The DR is centrally placed and excited using a stepped-impedance cross-slot aperture etched in the ground plane. The slot dimensions are F1 = 8 mm and F2 = 15.8 mm, with an additional stepped section of length F_l_ = 5.5 mm to enhance electromagnetic coupling. The detailed geometrical parameters are illustrated in Fig. 1 and listed in Table 2. The resonator is depicted in both top and 3D views to facilitate easy identification. The performance of the antenna, particularly its dual-band resonance and impedance matching, is strongly influenced by the geometry of the aperture slot etched in the ground plane. To determine the optimal configuration, a systematic design evolution was carried out, as illustrated in Fig. 2.

**Table 1:** Design evolution phases.

Phase	Slot geometry	Resonance behavior	Impedance matching
First	Rectangular slot	Poor resonance around target bands	Weak
Second	Uniform cross-slot	Dual-band; Shifted	Partial matching, Moderate
Third	Stepped-impedance cross-slot	Dual-band at 3.5 GHz & 3.9 GHz	Enhanced

As presented in Table 1, in the first phase, a conventional rectangular slot was used for aperture coupling; however, the S-parameter response showed poor impedance matching at the target frequencies of 3.5 and 3.9 GHz, resulting in weak resonance and low radiation efficiency. In the second phase, the slot was modified into a uniform cross shape, which established a dual-band response and improved impedance matching, but the lower resonance at 3.5 GHz remained misaligned, and the matching at 3.9 GHz was still suboptimal. The final, third phase employed a stepped-impedance cross slot, with the central portion wider than the arms, allowing precise control of current distribution along the slot. This design successfully excited the desired resonant modes—fundamental TE₁₁δ at 3.5 GHz and higher-order TE₁₂δ at 3.9 GHz while achieving excellent impedance matching (|S₁₁| < − 10 dB).

**Fig. 3:**
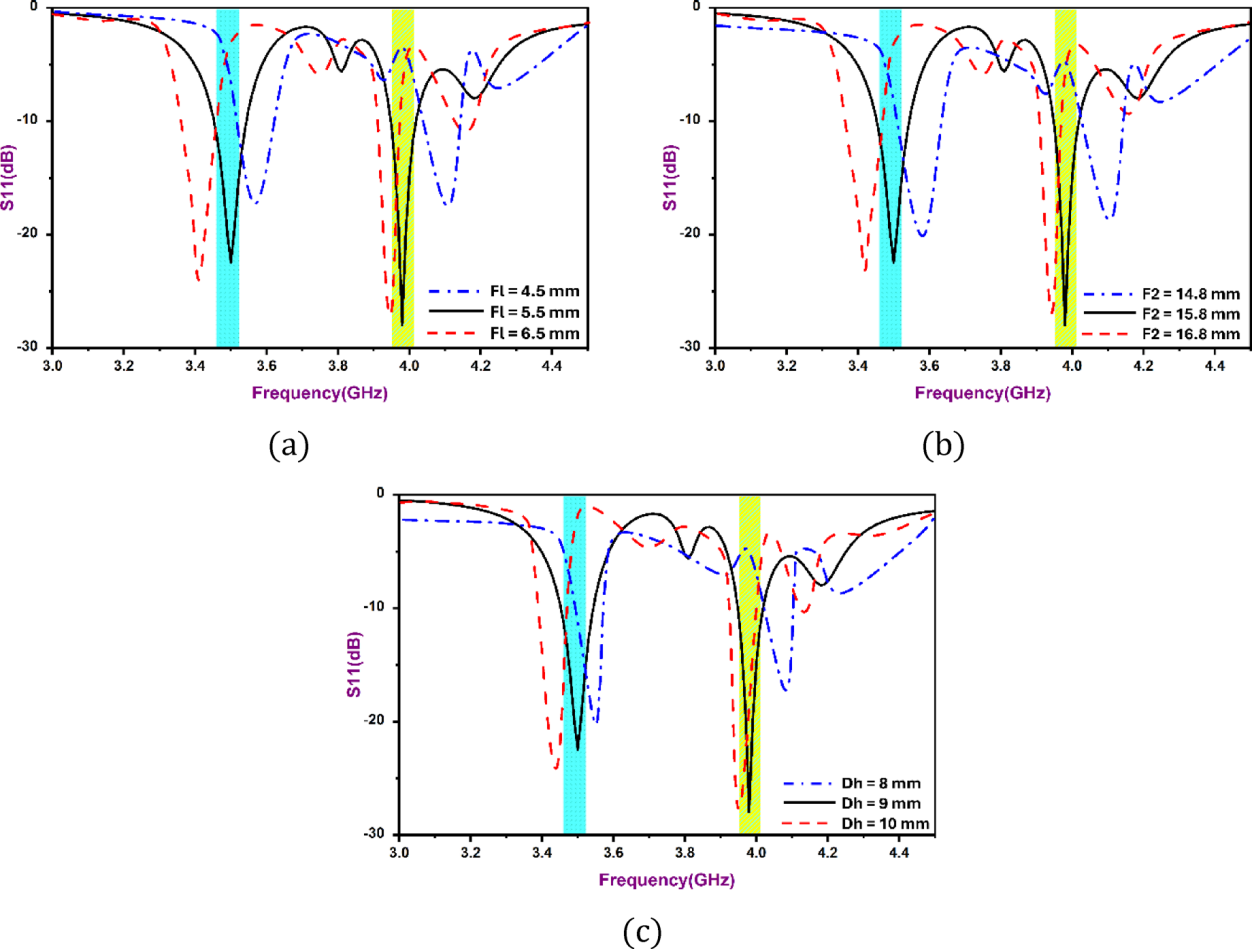
Simulated $\:{S}_{11}$for different values of (**a**) $\:{F}_{l}$, (**b**) $\:{F}_{2}$, and (**c**) $\:{D}_{h}$.

A parametric study was done to establish the effects of the geometrical design in the performance of the antenna. F_l_ (the length of the stepped-slot), F_2_ (the length of the cross-slot arm) and D_h_ (the height of the dielectric-resonator), were varied and all other parameters kept constant. As shown in Fig. 3a, increasing the size of F_l_ increases the coupling between the resonator and the feed. As a result, the impedance match is increased and there is a small shift in resonant frequency downwards. Figure 3b indicates that when F_2_ increases, the effective electrical length of the slot gets longer causing the resonant frequency to shift downward. Figure 3c shows that the larger D_h_ lowers the Q factor of the resonator thus is able to achieve a wider band of impedance.

Consequently, the optimized geometry delivered higher radiation efficiency, stable broadside E-plane and nearly omnidirectional H-plane patterns, strong inter-port isolation, and minimal cross-polarization, making it suitable for the final MIMO prototype. While the aperture slot influences surface current distribution, its primary function is controlled aperture coupling and mode excitation rather than acting as a dedicated decoupling structure.

**Table 2:** Geometrical specifications of the antenna element (mm).

Parameter	Dimensions (mm)	Parameter	Dimensions (mm)
Ws	30	Ls	30
Dx	11	Dy	14
Pw	25	Wm	90
Pl	19	Fl	5.5
Dh	9	t	1.56
R2	0.5	F2	15.8
R1	1	Lm	90
F1	8		

Each antenna element is roughly 30 mm by 30 mm in dimension, with an operational frequency of 3.5 GHz, corresponding to 0.35λ × 0.35λ. With the four elements arranged in a 2 × 2 layout and accounting for spacing between them, the total footprint of the MIMO system is approximately 65 mm × 65 mm, which supports compact integration in space-constrained Sub-6 GHz 5G devices. The geometrical specifications of single antenna element are depicted in Table 2. Although the overall footprint is 65 × 65 mm², the design remains electrically compact (approximately 0.75λ₀ × 0.75λ₀ at 3.5 GHz). For Sub-6 GHz 5G user equipment and indoor access points, spatial diversity requires adequate element spacing to maintain low correlation and high isolation. Therefore, the proposed size represents a practical trade-off between compactness and MIMO performance. The radiator occupies nearly one-fourth of the total area, while the remaining region ensures reduced mutual coupling and stable radiation characteristics. Hence, the configuration is optimized for performance-driven compact devices rather than ultra-miniaturized handheld platforms.

### Experimental findings with discussion

**Fig. 4:**
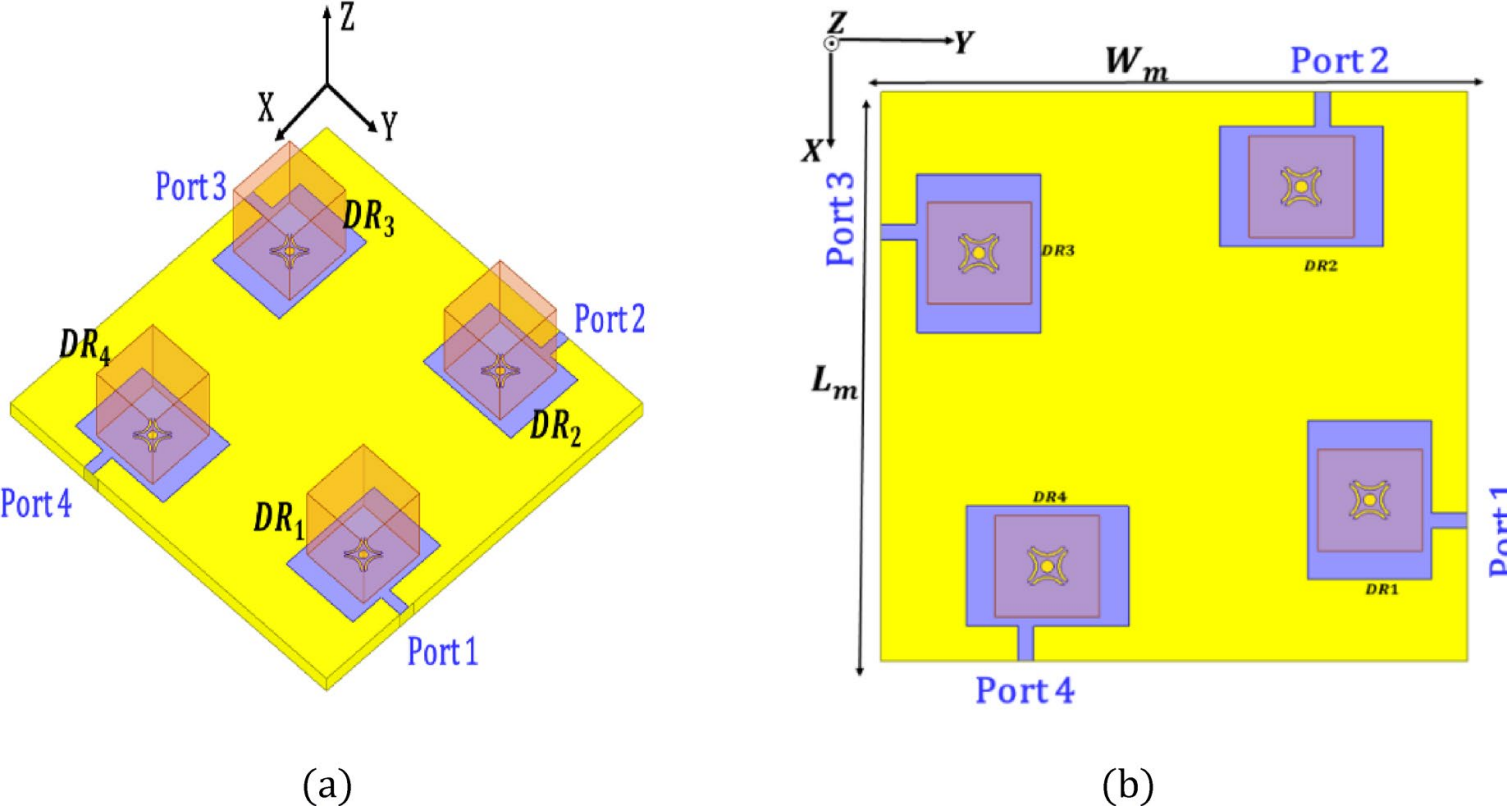
MIMO antenna (**a**) 3D (**b**) Top view.

Figure 4 depicts the MIMO antenna that has been designed. The quad-ports are physically separated from each other when they exit the four DRs.

**Fig. 5:**
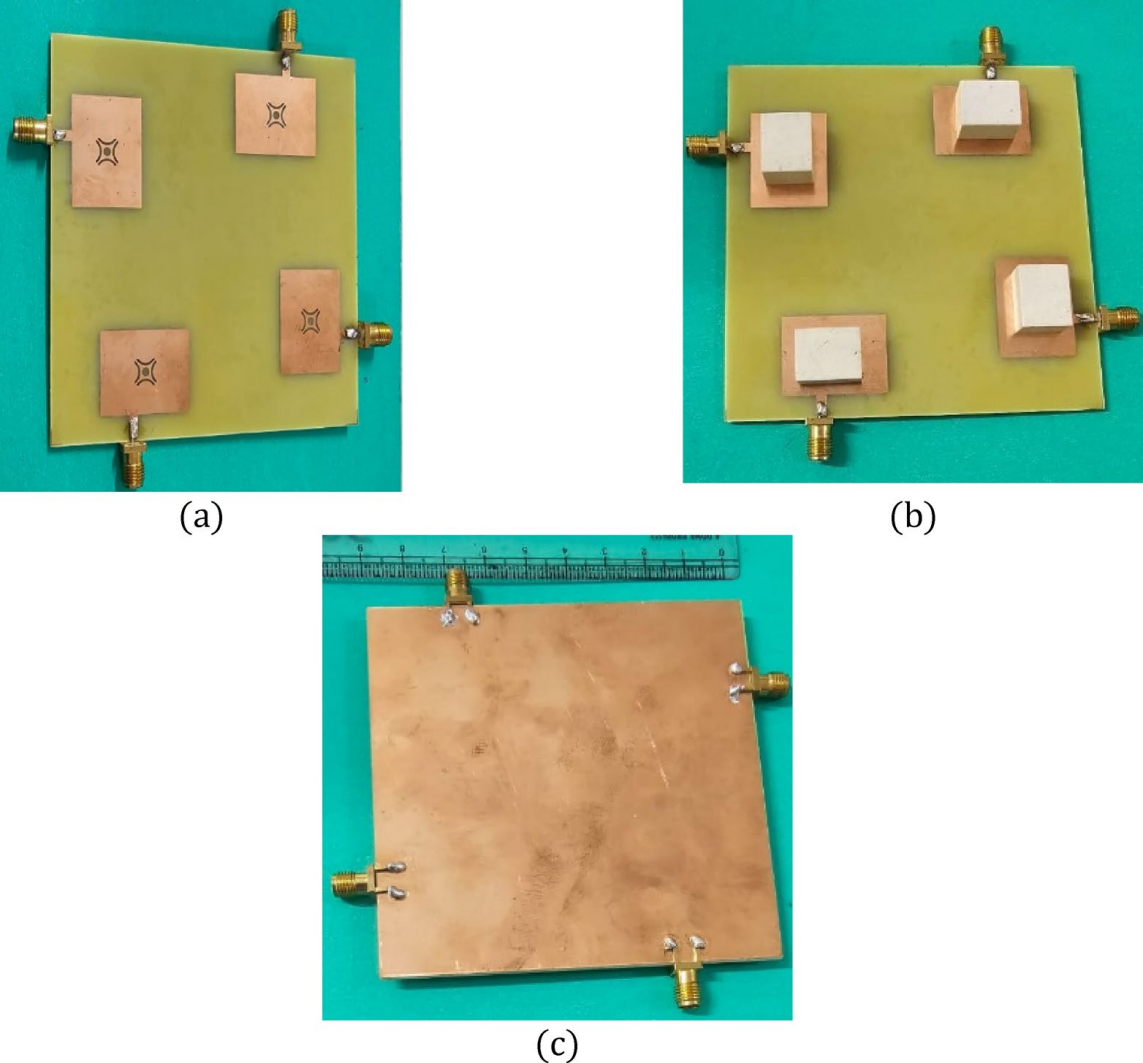
MIMO design (**a**) Configuration without dielectric resonators (DR), (**b**) Reference plane structure, and (**c**) Final configuration with integrated dielectric resonators (DR).

**Fig. 6:**
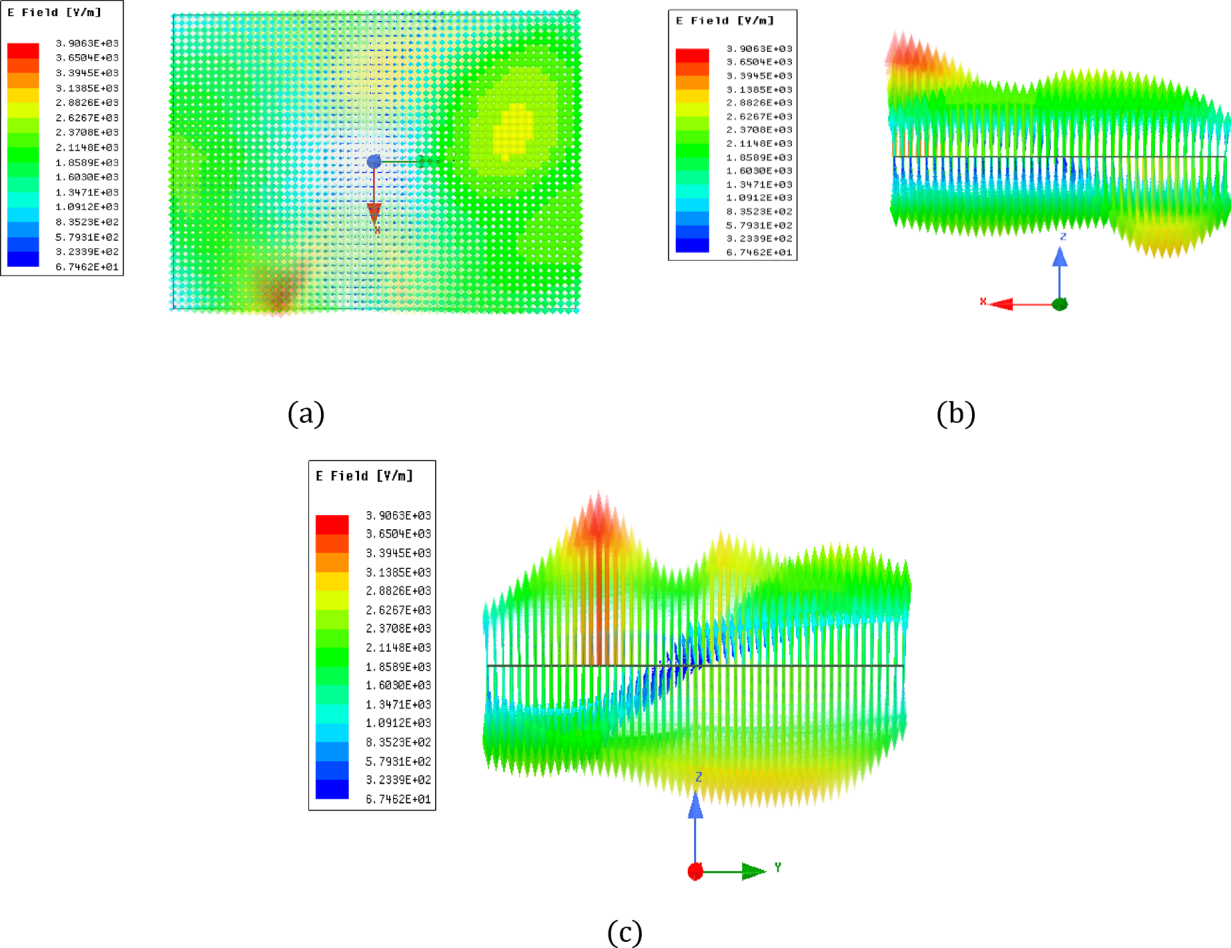
Simulated electric field distribution inside the dielectric resonator at 3.5 GHz illustrating excitation of the fundamental TE_11_δ mode: (**a**) top view, (**b**) right view, and (**c**) left view.

**Fig. 7:**
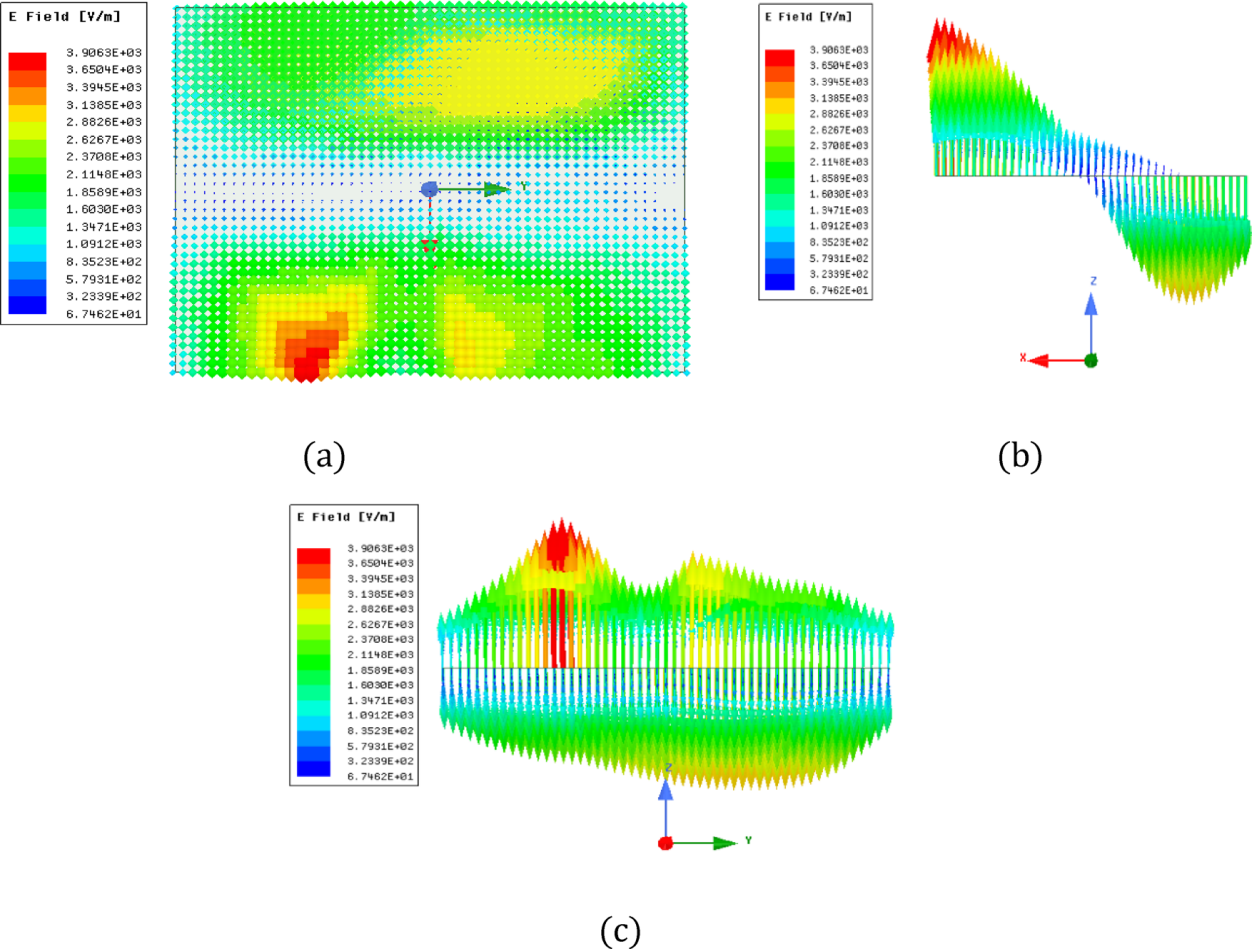
Simulated electric field inside the dielectric resonator at the resonant frequency of 3.9 GHz: (**a**) Upper view, (**b**) Factual view, (**c**) Left view.

Figure 5 illustrates the antenna prototype that was created using FR-4 laminate. Laser cutting was employed to shape the dielectric material according to the optimized dimensions. The antenna employs an FR-4 substrate characterized by εr = 4.4 & tan δ = 0.02 at 3.5 GHz, while the dielectric resonator material possesses εr = 9.8 and tan δ = 1 × 10⁻⁴ – 5 × 10⁻⁴. These values were considered during simulation and fabrication to ensure accurate representation of practical performance. To further validate the dual-band operation, the E field distributions inside the dielectric resonator were analyzed at the resonant frequencies. Figure 6 illustrates the simulated electric field distribution inside the dielectric resonator at 3.5 GHz. The field is predominantly oriented along a single principal direction, confirming excitation of the fundamental TE₁₁_δ_ mode with linear polarization characteristics. The field confinement within the resonator supports stable fundamental-mode resonance at this frequency. At 3.9 GHz, the antenna excites a higher-order TE₁₂δ resonant mode exhibiting linear polarization characteristics. The field plots clearly show the higher-order field variations along the resonator, which are characteristic of this mode, as shown in Fig. 7. The TE₁₂δ mode at 3.9 GHz is excited due to the stepped-impedance cross-slot aperture, which enhances coupling and promotes higher-order field variations within the dielectric resonator. The electric field distribution at 3.9 GHz shows multiple field maxima characteristic of TE_12_δ behavior, while the corresponding surface current patterns further confirm this higher-order resonance. This validates the origin of the second operating band.

**Fig. 8:**
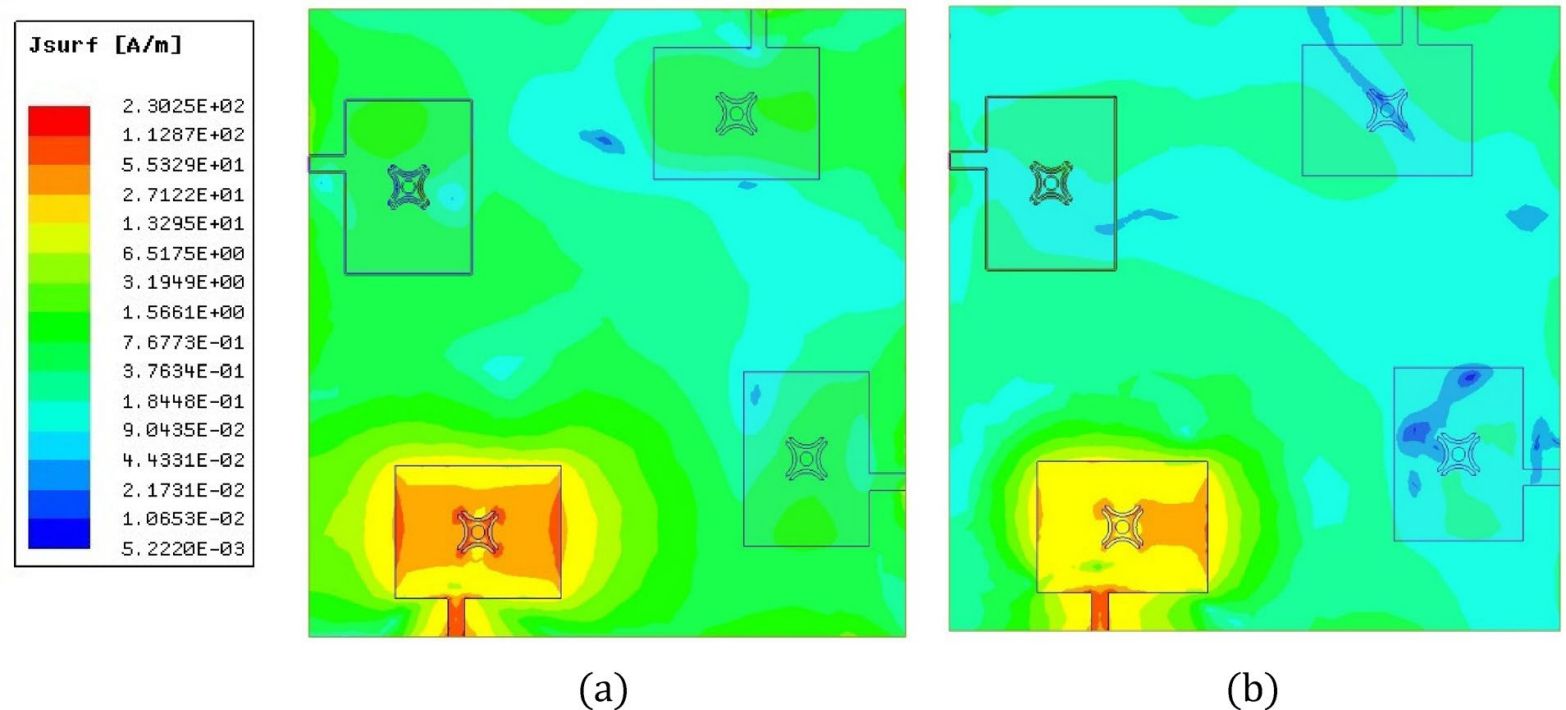
Surface current distribution on the ground plane and feeding structure at (**a**) 3.5 GHz and (**b**) 3.9 GHz.

To further clarify the dual-mode operation, the surface current distributions on the ground plane and feeding structure were analyzed at 3.5 GHz and 3.9 GHz, as shown in Fig. 8. At 3.5 GHz, the surface currents are primarily concentrated along a dominant direction on the resonator surface, consistent with excitation of the fundamental TE₁₁δ mode exhibiting linear polarization characteristics. At 3.9 GHz, the current distribution shows increased spatial variations and additional current maxima along the resonator structure, confirming excitation of a TE₁₂δ mode. The distinct current profiles at the two frequencies verify the dual-mode resonance responsible for the antenna’s dual-band operation.

**Fig. 9:**
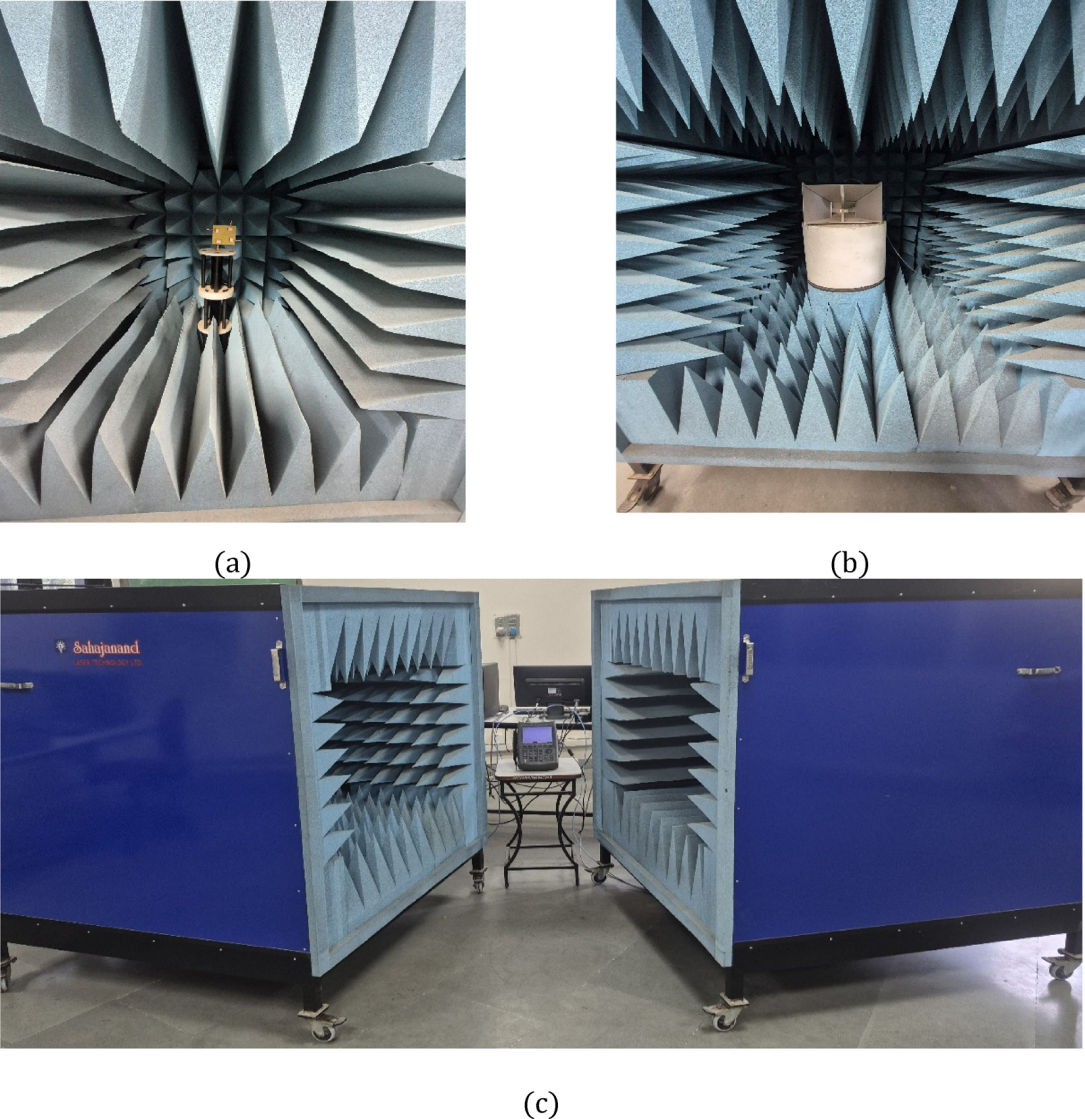
(**a**) MIMO antenna (**b**) Horn antenna (**c**) Laboratory setup.

**Fig. 10:**
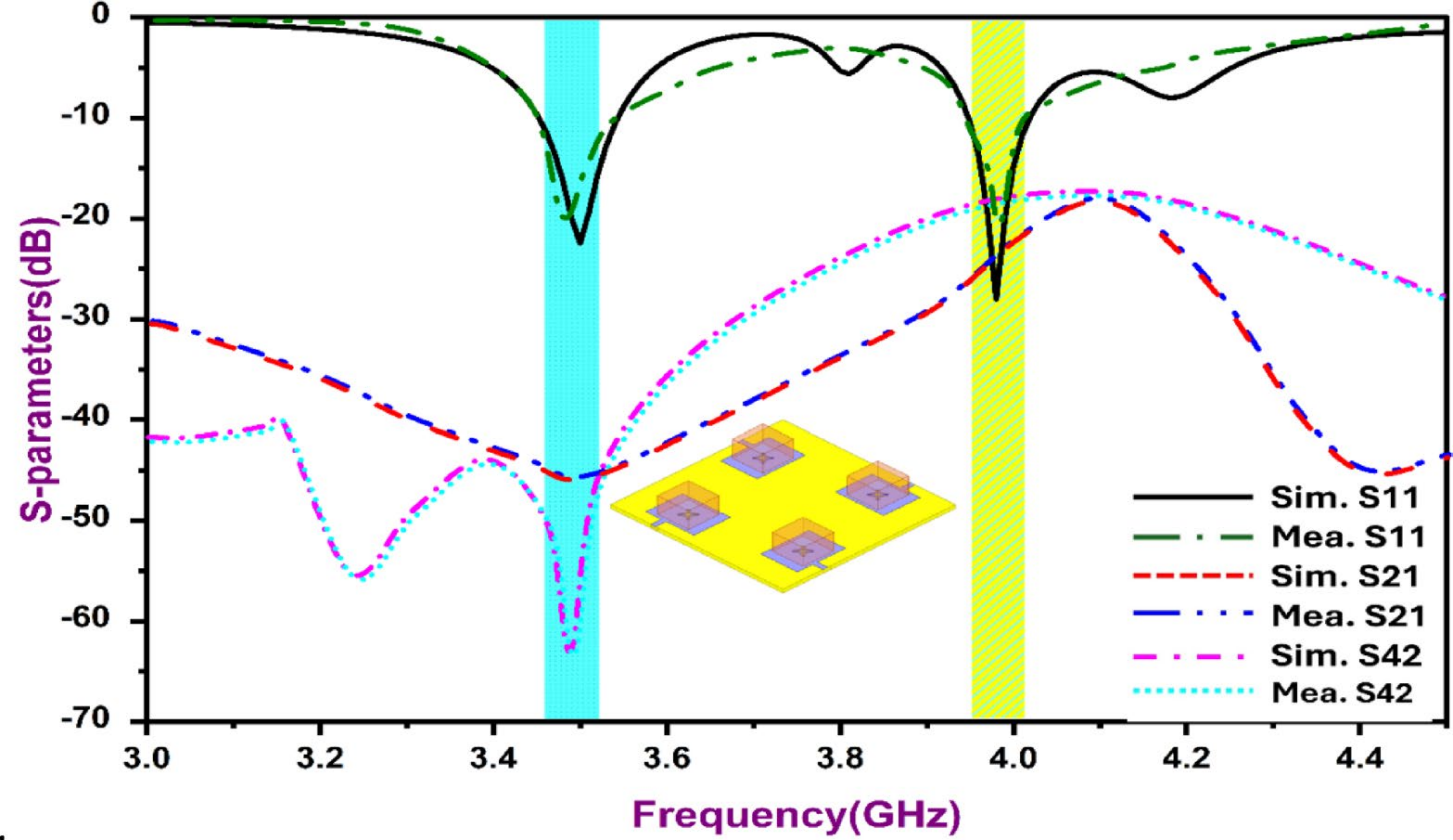
S-parameters of the planned four-port DRA MIMO arrangement.

Figure 9a exhibits the MIMO antenna mounted inside the anechoic chamber. The chamber has dimensions of 3ft x 3ft x 3ft and is able to provide isolation up to 25 dB. The horn antenna shown in Fig. 9b has a frequency range from 900 MHz to 18 GHz, covering the targeted frequencies using the presented MIMO designs. The full laboratory setup, having open-ended chambers, is exhibited in Fig. 9c along with the VNA setup. At the time of radiation pattern measurement, the chamber is closed to ensure full isolation. The fabricated antenna was tested using an N9912A Vector Network Analyzer (VNA). The measured and simulated S-parameters of the quad-port antenna are shown in Fig. 10, confirming dual-band resonance at 3.5 GHz and 3.9 GHz. The achieved isolation (> 20 dB) results from the combined effects of modal diversity, spatial separation between antenna elements, and electromagnetic field confinement within the dielectric resonator. The dielectric resonator confines electromagnetic fields within its volume, thereby suppressing surface-wave propagation and reducing ground-plane current coupling compared to conventional patch antennas. At 3.5 GHz and 3.9 GHz, distinct resonant modes (TE₁₁δ and TE₁₂δ) are excited, each exhibiting spatially confined field distributions. This modal distinction further minimizes near-field overlap between adjacent antenna elements, contributing to improved inter-port isolation.

**Fig. 11:**
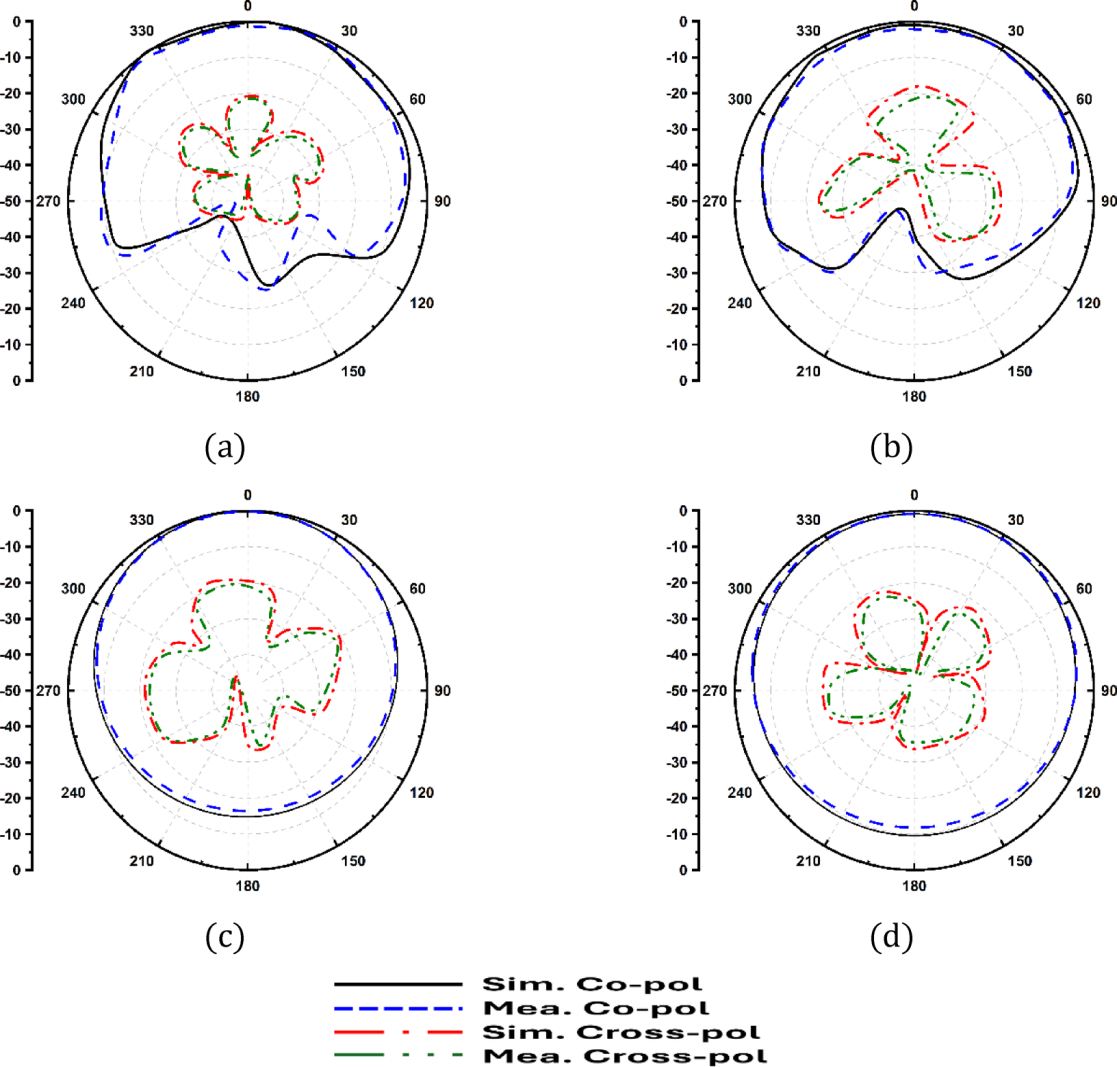
Radiation patterns: (**a**) E-plane @ 3.5 GHz, (**b**) E-plane @ 3.98 GHz, (**c**) H-plane @ 3.5 GHz, and (**d**) H-plane @ 3.98 GHz.

**Fig. 12:**
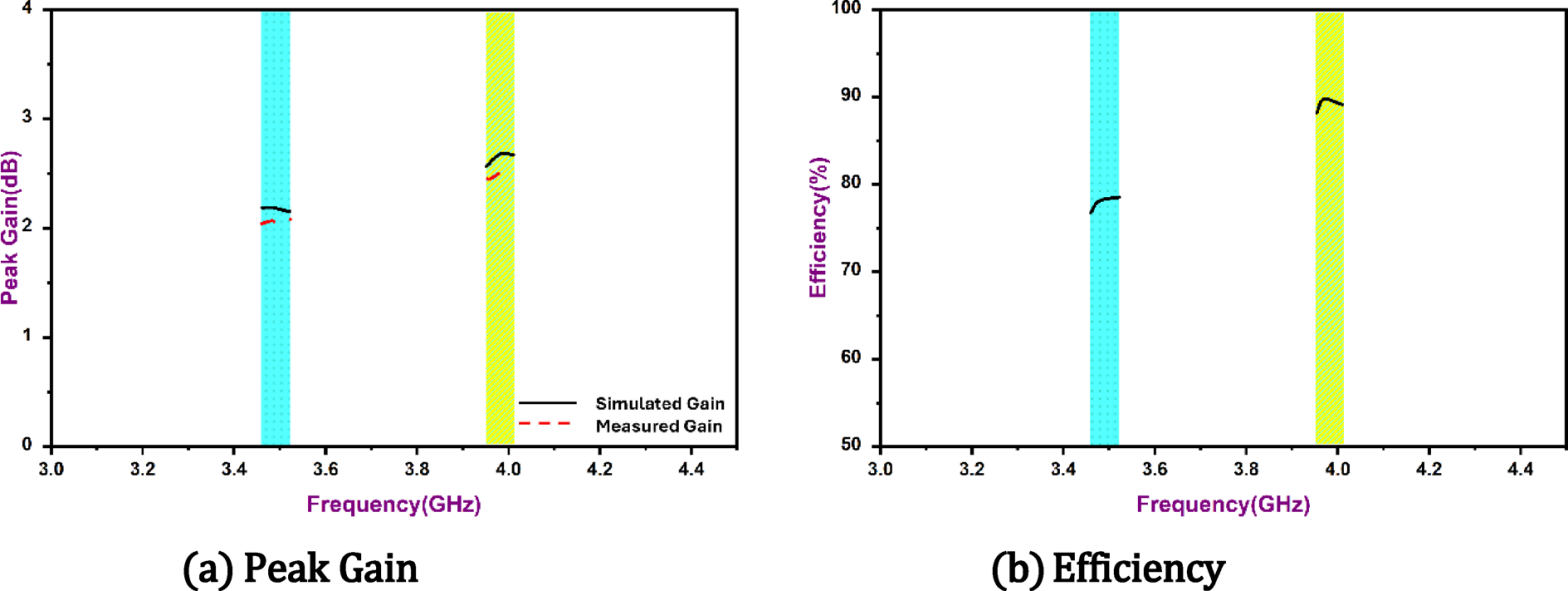
Gain and radiation efficiency of the proposed antenna.

Furthermore, the 2 × 2 spatial configuration provides adequate inter-element spacing to suppress surface current interaction across the ground plane. The aperture-fed configuration further reduces direct current leakage, enabling intrinsic isolation without external decoupling structures Radiation patterns at both resonances (Fig. 11) confirm stable broadside E-plane and nearly omnidirectional H-plane characteristics, with cross-polarization levels at least 15 dB below the co-polarized components. The gain and radiation efficiency of the proposed antenna across the operating frequency range are shown in Fig. 12. The antenna achieves a peak gain of approximately 2.6 dBi with radiation efficiency approaching 89% at the operating bands.

## MIMO diversity parameters

**Fig. 13:**
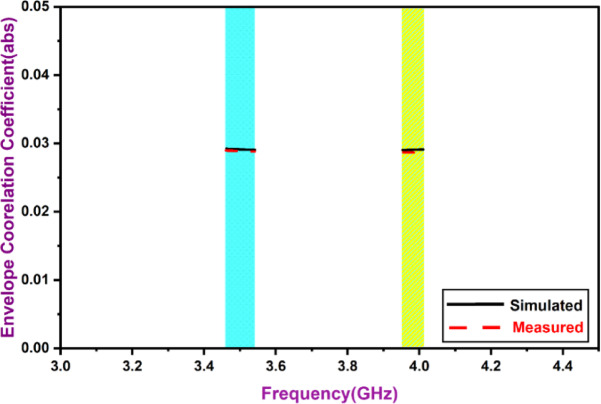
MIMO ECC.

Communication over wireless networks creates an unfriendly atmosphere. Through diversity techniques, MIMO communication is able to boost spectral efficiency. The diversity contributes to an increase in the reliability of communication. The communication efficiency will be increased by sending the same information chunk across multiple, separate, and independent channels, allowing for uneven fading over them. The utilization of several antennas in MIMO configurations is an appropriate method for achieving such spatial variety (Fig. 13).

The envelope correlation coefficient quantifies the correlation between radiation patterns^1,25^. Horizontal and vertical polarization patterns must be uncorrelated, ideally yielding a coefficient of zero, as the radiation is directed oppositely to the MIMO elements.

1 $$\:\rho\:e=ECC=\:\frac{\begin{array}{c}\left|\underset{0}{\overset{4\pi\:}{\iint\:}}\left[\overrightarrow{\boldsymbol{F}1}\:\left(\theta\:,\:\varnothing\:\right)*\:\overrightarrow{\boldsymbol{F}2}\:\left(\theta\:,\:\varnothing\:\right)\right]d\varOmega\:\right|\\\:0\end{array}}{\underset{0}{\overset{4\pi\:}{\iint\:}}\left|\overrightarrow{\boldsymbol{F}1}\:\left(\theta\:,\:\varnothing\:\right)\right|\mathrm{2}d\varOmega\:\underset{0}{\overset{4\pi\:}{\iint\:}}\left|\overrightarrow{\boldsymbol{F}2}\:\left(\theta\:,\:\varnothing\:\right)\right|\mathrm{2}d\varOmega\:}$$

**Fig. 14:**
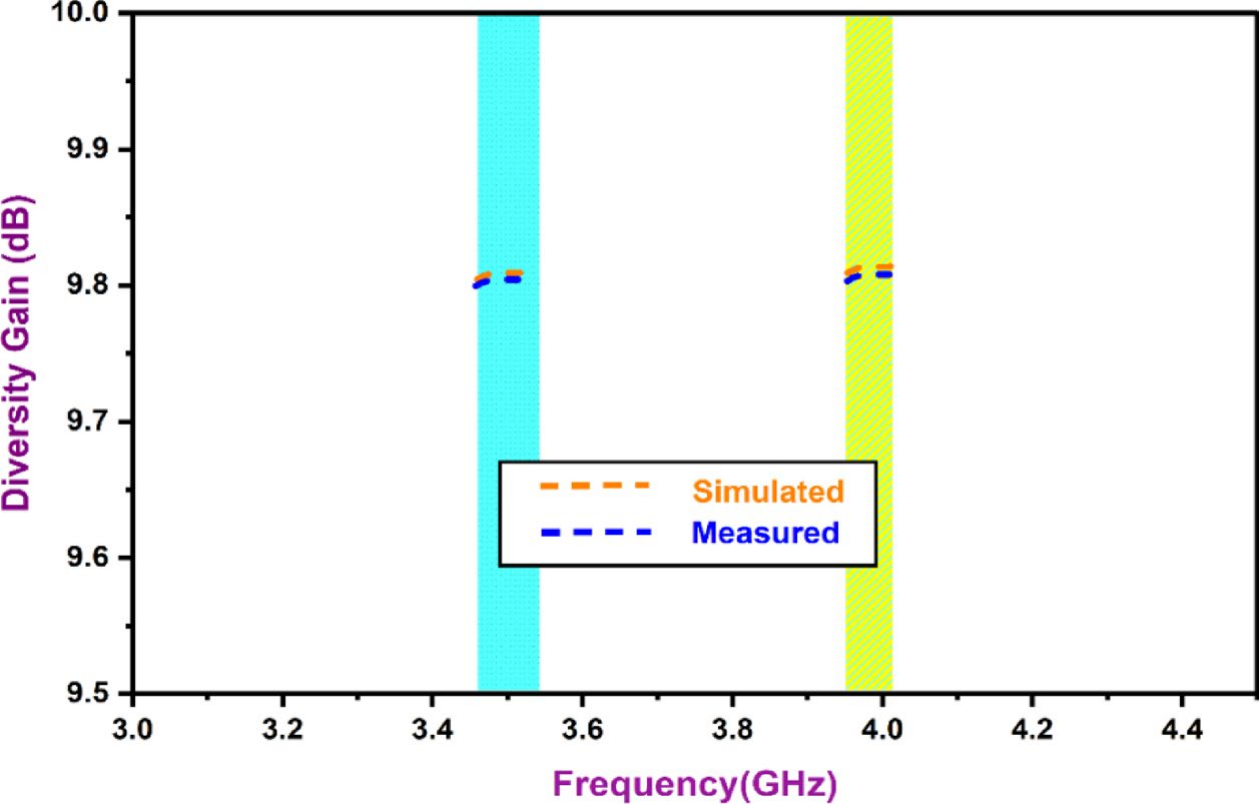
MIMO DG.

Where *R*_*i*_*(θ*,* φ)* and *R*_*j*_*(θ*,* φ)* are three-dimensional patterns that represent two sequential resonators, i and j, respectively. The solid angle measures *ω*, the Hermitian product is *, *θ* equal to elevation angle & azimuth angle is denoted by *φ*. According to Fig. 13, the values of ECC that are commonly anticipated to be present are often lower than 0.05. The antenna that was designed does have a range of ECC that is adequate. A representati3on of the directivity gain (DG) can also be found in Fig. 14. Through the use of spatial diversity, MIMO communication is able to resolve multipath signals, effectively resulting in a diversity gain. It retrieves data from several independent channels that are experiencing fading because of unanticipated channel conditions, resulting in a significant improvement in the system’s intelligence. A calculation of the DG can be made by:

2 $$\:DG=10\sqrt{1-{\left|0.99\:ECC\right|}^{2}}$$

A crucial indicator for assessing the efficiency of MIMO systems is, mean effective gain (MEG), which quantifies received power fraction relative to isotropic antenna, taking into account the various channel characteristics. The MEG is presented as:

3 $$\:{MEG}_{i}=0.5\left[1-\sum\:_{j=1}^{N}{\left|{S}_{ij}\right|}^{2}\right]$$

**Fig. 15:**
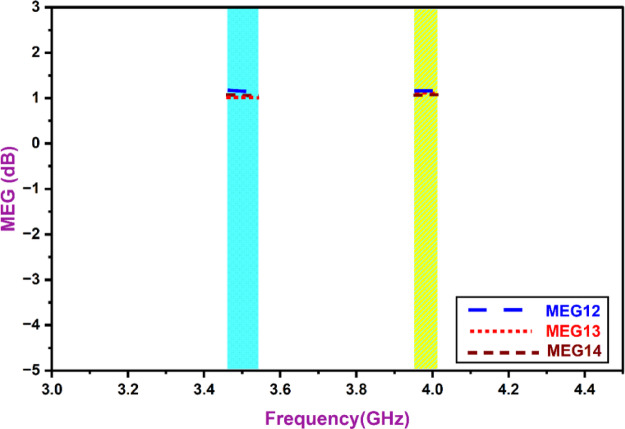
MIMO MEG.

The exceptionally high power ratio is caused by the sparse positioning and reduced mutual interaction between the components of the antenna. Figure 15 indicates the MEG energy ratios of the 4-port antenna.

**Fig. 16:**
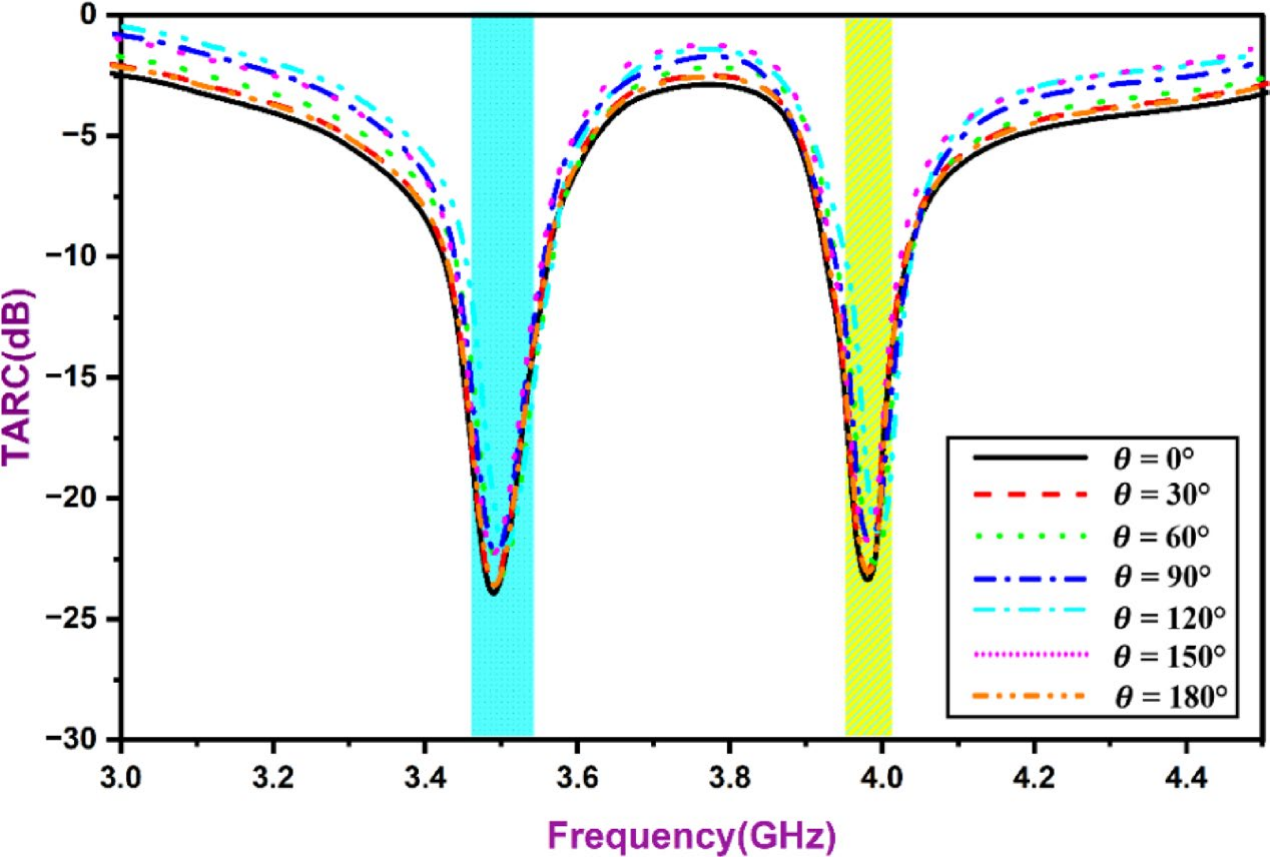
MIMO TARC.

In addition, an analysis was conducted to accurately describe the MIMO system by examining efficiency, MEG, CCL (channel capacity loss), ECC, TARC (total active reflection coefficient), &DG. TARC calculates proportion of power reflection to incident power, while CCL determines the highest rate of data transfer at which a continuous, error-free transmission of the information stream is possible. The optimum value of TARC is -22, as depicted in Fig. 16. The TARC is obtained by applying the formula is given in reference^8^:

4 $$\:TARC=\frac{\sqrt{{\sum\:}_{n=1}^{N}{\left|{b}_{n}\right|}^{2}}}{\sqrt{{\sum\:}_{n=1}^{N}{\left|{a}_{n}\right|}^{2}}}$$

5 $$\:{b}_{n}=\left[S\right]{a}_{n}$$

**Fig. 17:**
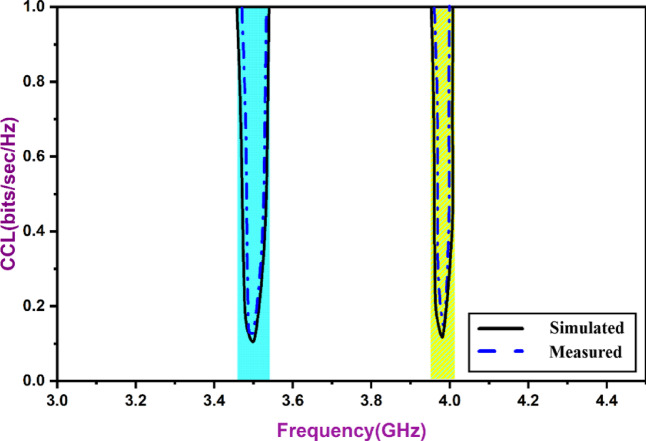
MIMO CCL.

The CCL (Channel Capacity Limit) represents the maximum data transfer rate in bits per second per hertz that can be achieved without significant loss. For a well-designed MIMO structure, CCL value is close to 0.4 bits/s/Hz, as stated in reference^2,9^. The evaluation can be performed using Eq. (6). The suggested MIMO antenna exhibits reduction in channel capacity of 0.1 bits/s/Hz, confirming an improved data transmission rate without the need for additional power. Figure 17 illustrates the suggested antenna system’s Channel Capacity Loss characteristics, demonstrating a low score of 0.1 bits/s/Hz, which meets the requirement for efficient MIMO systems.

6 $$\:CCL=-{log}_{2}\:det\left({{\Psi\:}}^{R}\right)$$

7 $${{\psi }}^{R} = \left[ {\begin{array}{*{20}c} {\rho _{{11}} } & {\rho _{{12}} } & {\rho _{{13}} } & {\rho _{{14}} } \\ {\rho _{{21}} } & {\rho _{{22}} } & {\rho _{{23}} } & {\rho _{{24}} } \\ {\rho _{{31}} } & {\rho _{{32}} } & {\rho _{{33}} } & {\rho _{{34}} } \\ {\rho _{{41}} } & {\rho _{{42}} } & {\rho _{{43}} } & {\rho _{{44}} } \\ \end{array} } \right]$$

8 $$\rho _{{ii}} = 1 - \sum\limits_{{n = 1}}^{4} {\left( {S_{{in}} * S_{{ni}} } \right)} ;\,\rho _{{ij}} = - \sum\limits_{{n = 1}}^{4} {\left( {S_{{in}} * S_{{nj}} } \right)\,} For\,i,j = 1,2,3,or{\text{ }}4$$

## Performance benchmarking

Table 3 provides the benchmarking of the proposed aperture-fed quad DR-MIMO antenna with existing Sub-6 GHz MIMO designs. Unlike^10,11,15^, which require additional ground modifications or decoupling structures to achieve isolation in the range of 14–17 dB, our antenna achieves inter-port isolation exceeding 20 dB at the operating bands through a combination of spatial arrangement and modal diversity. The ECC of the proposed design (< 0.05) is substantially less than 0.06–0.12 range reported in^10–13,19^, leading to superior diversity performance. Furthermore, the achieved CCL of only 0.1 bps/Hz is significantly smaller than those in^10–12,19^, which lie in the 0.28–0.35 bps/Hz range. Compared to^13,16^, which employ larger or more complex AMC and cup-shaped geometries, the proposed antenna maintains a compact footprint of 65 × 65 mm² while still offering dual-band operation at 3.5/3.9 GHz. In^17^, although good gain (2.5 dBi) and isolation (18 dB) are achieved, the design uses a more complex Y-fed structure and suffers from much higher CCL (2.5 bps/Hz). Similarly^19^, employs a rhombic feed structure but reports higher ECC (0.07) and larger CCL (0.33 bps/Hz). Importantly, the simultaneous excitation of fundamental TE11δ and higher-order TE₁₂δ modes within a compact quad-port configuration without additional decoupling structures is not commonly reported in earlier works marking a key novelty of this study. Compared to the closest related works^17,19^, our design simultaneously provides higher isolation (> 20 dB vs. 17–18 dB) and significantly lower CCL (0.1 bps/Hz vs. 0.33–2.5 bps/Hz), while still maintaining a compact 65 × 65 mm² footprint. This balance of performance and size is not reported in prior literature.

**Table 3:** MIMO antenna performance evaluation for sub-6 GHz use cases.

Ref	Antenna type	Isolation (db)	ECC	Gain (dBi)	CCL (bps/Hz)
^10^	Inverted-F MIMO	15	0.12	1.9	0.35
^11^	L-shaped monopole MIMO	17	0.08	2.1	0.28
^12^	Arc-shaped MIMO	16	0.07	2.3	0.30
^13^	8-element PDA with AMC	18	0.06	2.4	NR
^14^	Uniplanar quad-port MIMO	14	0.09	2.2	0.27
^15^	Hex-ring MIMO	16	0.11	1.8	NR
^16^	Cup-shaped DRA MIMO	17	0.06	2.4	0.30
^17^	Composite Y-fed DRA	18	0.05	2.5	2.5
^19^	Rectangular DRA with rhombic feed	17	0.07	2.1	0.33
This work	Aperture-Fed Quad DR-MIMO	> 20	< 0.05	2.62	0.1

Isolation values are reported at the respective operating frequencies.

## Conclusion

The design, fabrication, and experimental validation of a compact quad-port dielectric resonator MIMO antenna for sub-6 GHz 5G applications were presented. Although the peak gain is moderate, it is well suited for compact user terminals and indoor 5G devices, where omnidirectional coverage and low correlation are prioritized over high directivity. The antenna employs a multi-mode excitation strategy that combines a fundamental TE₁₁_δ_ mode at 3.5 GHz with a higher-order TE₁₂_δ_ mode at 3.9 GHz. Along with an optimized spatial arrangement, this approach effectively reduces mutual coupling and achieves intrinsic inter-port isolation exceeding 20 dB without the need for separate parasitic decoupling networks. Experimental results demonstrate excellent diversity performance with ECC below 0.05, CCL of approximately 0.1 bps/Hz, and high radiation efficiency. The close agreement between simulated and measured results confirms the robustness of the proposed design. Overall, the antenna provides a practical solution for compact sub-6 GHz MIMO systems requiring high isolation and reliable diversity performance. The antenna demonstrates stable dual-band linear polarization with intrinsic isolation exceeding 20 dB without additional decoupling structures.

## Data Availability

The data used to support the findings of this study are included in the article.

## Declarations

### **Declaration of AI and AI-Assisted Technologies in the Writing Process:**

During the preparation of this manuscript, the authors used Grammarly/AI-assisted language tools to improve grammar, spelling, and language clarity. The authors carefully reviewed and edited the output and take full responsibility for the content of the publication.
